# Polyphenols–Gut–Heart: An Impactful Relationship to Improve Cardiovascular Diseases

**DOI:** 10.3390/antiox11091700

**Published:** 2022-08-30

**Authors:** Francesca Bianchi, Annalisa Cappella, Nicoletta Gagliano, Lucia Sfondrini, Alessandra Stacchiotti

**Affiliations:** 1 Department of Biomedical Sciences for Health, Università degli Studi di Milano, Via Mangiagalli 31, 20133 Milan, Italy; 2 U. O. Laboratorio Morfologia Umana Applicata, IRCCS Policlinico San Donato, San Donato Milanese, 20097 Milan, Italy; 3 Molecular Targeting Unit, Department of Research, Fondazione IRCCS Istituto Nazionale dei Tumori, 20133 Milan, Italy

**Keywords:** gut microbiome, polyphenols, atherosclerosis, hypertension, coronary artery disease

## Abstract

A healthy gut provides the perfect habitat for trillions of bacteria, called the intestinal microbiota, which is greatly responsive to the long-term diet; it exists in a symbiotic relationship with the host and provides circulating metabolites, hormones, and cytokines necessary for human metabolism. The gut–heart axis is a novel emerging concept based on the accumulating evidence that a perturbed gut microbiota, called dysbiosis, plays a role as a risk factor in the pathogenesis of cardiovascular disease. Consequently, recovery of the gut microbiota composition and function could represent a potential new avenue for improving patient outcomes. Despite their low absorption, preclinical evidence indicates that polyphenols and their metabolites are transformed by intestinal bacteria and halt detrimental microbes’ colonization in the host. Moreover, their metabolites are potentially effective in human health due to antioxidant, anti-inflammatory, and anti-cancer effects. The aim of this review is to provide an overview of the causal role of gut dysbiosis in the pathogenesis of atherosclerosis, hypertension, and heart failure; to discuss the beneficial effects of polyphenols on the intestinal microbiota, and to hypothesize polyphenols or their derivatives as an opportunity to prevent and treat cardiovascular diseases by shaping gut eubiosis.

## 1. Introduction

The human intestine is considered an endocrine organ, able to communicate locally with resident commensal bacteria, called the gut microbiota (GM), and indirectly with distal organs by producing metabolites, toxins, and inflammatory mediators in the blood circulation [1,2]. The GM has been defined as “the last undiscovered human organ” due to its crucial influence on the host metabolism and immunity and the intricate relationships with other fundamental organs, such as the brain and heart [3,4,5].

Preclinical and clinical studies demonstrate that the prevalence of detrimental gut bacteria and their inflammatory metabolites may be a precursor to the onset and progression of local colorectal cancer and other distal tumors [6,7,8,9], neurodegenerative disorders [10,11,12,13,14], primary osteoporosis [15], and cardiovascular diseases (CVDs) [16,17,18]. Recent epidemiological data by WHO European Region clearly indicate that CVDs, mainly ischemic heart disease and stroke, are the most common causes of death [19]. The Global Injuries and Risk Factors analysis has examined ten years of data (from 2009 to 2019) on mortality in different world areas, confirming the prevalence of cardiovascular mortality in China, followed by India, the Russian Federation, the United States, and Indonesia [20]. Moreover, the COVID-19 pandemic is also associated with acute and post-acute cardiovascular and thromboembolic manifestations in survivors [21,22]. Excluding genetic risk, the most common modifiable risk factors of CVDs are an unhealthy lifestyle [23], smoking [24], alcohol consumption [25], and a hypercaloric diet [26]. Remarkably, the shift to a diet based on fiber-rich whole grains, fish, poultry, vegetables, and fruit greatly prevents and ameliorates the outcomes of CVDs [27,28]. Polyphenols are heterogeneous plant-derived molecules, highly present in vegetables, wine, cereals, tea, and coffee, and diffuse in human nutrition [29]. Preclinical studies indicate the potential role of polyphenols as prebiotics able to modulate intestinal permeability and GM composition, but their effect in clinical studies is still debated [30,31,32]. Polyphenols may be effective to prevent CVDs and ensuring cardiometabolic health [33,34,35].

In this review, we firstly focus on the anatomy of the small and large intestine; then, we discuss in vitro and in vivo studies on the relationship between the GM and CVDs; finally, we analyze the dietary bioavailability of polyphenols, their argued bio-action in preventing CVDs, and their potential mechanisms to restore GM homeostasis; notably, we critically discuss the potential use of polyphenols as a novel strategy for the improvement of CVDs via intestinal bacterial modulation.

## 2. The Small and Large Intestine: Different Structures and Functions

**Figure 1:**
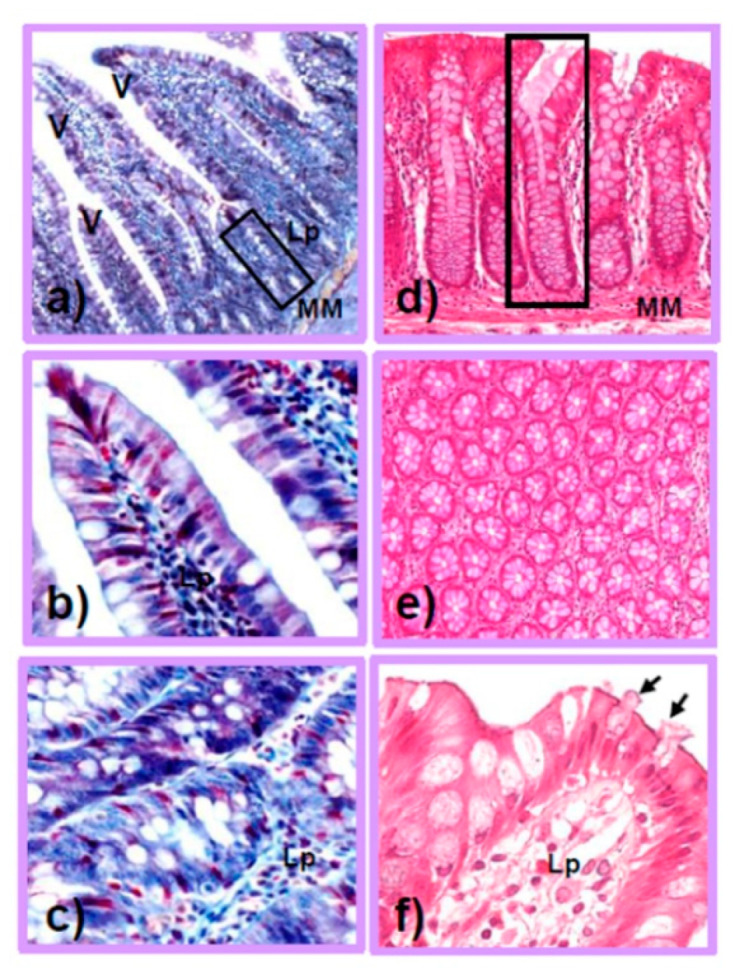
Representative micrographs of small intestine (jejunum) (**a**,**c**,**e**) and large intestine (colon) (**b**,**d**,**f**). (**a**) Jejunum: tunica mucosa showing the villi (V) projecting into the lumen and the intestinal Lieberkühn crypts extending in the lamina propria (Lp) (some of those in the boxed area are enlarged in panel **c**). The muscularis mucosae (MM) is also observed. (**b**) Villus lined by a simple columnar epithelium, including the enterocytes (stained in blue) and mucus-secreting goblet cells, with pale cytoplasm. The core of the villus is formed by the lamina propria (Lp). (**c**) Jejunum: at higher magnification, the crypts, located in the lamina propria (Lp) of the mucosa, contain abundant goblet cells. (**d**) Colon: tunica mucosa, showing the absence of villi and presence of abundant simple tubular glands, containing mostly goblet cells. (**e**) Cross-section of the simple tubular glands located in the connective tissue of the lamina propria in the mucosa of the colon. (**f**) Tunica mucosa of the colon, showing the simple columnar lining of the epithelium and the frequent goblet cells intercalated between the enterocytes—some of them are releasing their secretory products (arrows). They lie on the lamina propria (Lp). (**a**–**c**) Azan–Mallory staining; (**d**,**e**) H&E staining. Original magnification: 10× (**a**), 20× (**d**,**e**), 40× (**b**,**c**,**f**).

The human intestine is a hollow tubular organ, crucial for nutrient absorption and fecal transit, organized into the small and large intestines. The small intestine is microscopically characterized by mucosal finger-like projections called villi (Figure 1a,b). These unique protrusions amplify the surface for nutrient absorption, mucus secretion, and hormone release and are considered to play a primary role in absorption and digestion [36]. Even if enterocytes, characterized by highly oxidative metabolism, represent more than 80% of the intestinal mucosal cells [37], in the villi, there are also goblet cells, and rare tuft cells, taste-chemosensory cells, involved in the immune response [38]. The epithelial cells are sealed together by tight junctions (TJs), crucial for the intestinal barrier’s integrity and paracellular transport [39]. TJs are composed of specific transmembrane proteins called claudins, occludins, and intraplaque components forming the *zonula occludens*, necessary for the selective transport of nutrients, regulated by metabolites produced by probiotic bacteria. Lastly, enterocytes express a pattern of receptors, such as Toll-like receptors (TLRs), to recruit and activate inflammatory cells, and they are specific to each intestinal tract [40].

Remarkably, several cells of the innate immunity reside in the lamina propria and in the crypts of Lieberkühn, rich in intestinal stem cells (ISCs) [41,42], goblet cells, and Paneth cells (Figure 1c). In Paneth cells, both glycolytic activity and the production of lactate are pivotal for the proper differentiation of the intestinal barrier elements in the healthy gut [43,44,45]. Goblet mucus-secreting cells produce glycoproteins, such as mucins, essential for the luminal barrier, which senses and transports antigens in the intestinal immune tolerance system [46,47].

Lymphocytes are inserted in different layers in the wall of the small intestine, predominantly in the submucosa layer as isolated follicles or aggregates, called the Peyer patches (PPs) [48]. These formations, present mainly in the distal ileum, belong to gut-associated lymphoid tissues (GALTs), involved in the adaptive immune response to commensal bacteria [49]. In contrast with lymph nodes, PPs are devoid of a capsule and rich in germinal centers, signs of a rapid response to luminal antigens.

In the small intestine, the greatest number of commensal bacteria are localized in the human ileum, approximately 10^7^–10^8^ (CFU)/mL of the digesta; the most common are *Lactobacillus*, *Clostridium*, *Staphylococcus*, *Streptococcus*, and *Bacteroides* species, which are necessary for vitamin B12 and K storage, and for the degradation of carbohydrates and bile acids (BAs) [50].

In the duodenum, the first tract of the small intestine, the BA mixture is shaped by the bacterial metabolism, but, reciprocally, biliary salts influence the size of Gram-positive resident bacteria [51].

The large intestine, divided into the cecum, colon, and rectum segments, is approximately 1.8 m in length, with a diameter of 7 cm, and is morphologically characterized by the absence of villi (Figure 1d). The colon mucosa reabsorbs water, via transmembrane aquaporins and electrolytes, to form solid stools [52]. Indeed, the mucosa is lined by a single columnar epithelium, composed of enterocytes, called colonocytes, and goblet cells that reach approximately 25% of the luminal population in the distal colon [53]. This population is also prevalent in colonic crypts that are devoid of Paneth cells (Figure 1d,e). The high number of goblet cells in the colon, which intercalate between both the colonocytes (Figure 1f) and in the crypts (Figure 1d,e), is responsible for the production of a viscous layer acting as a lubricant for fecal transit, but also a chemical barrier to pathogens, a source of carbohydrates for beneficial commensal bacteria, or a matrix for antimicrobial molecules [54]. Recent studies indicate a direct active role of goblet cells in the transit of antigens to underlying immune cells and the modulation of host–bacteria interactions [55]. In contrast to the small intestine, which comprises a single layer of mucus, the distal colon contains a thick mucus layer, organized into two components: an outer compartment permeable to commensal bacteria and an inner sterile compartment. The chemical composition and abundance of mucins in the colon are modulated by resident bacteria and by the diet, leading to great inter-individual diversity. Indeed, a low-fiber Western diet causes bacteria-mediated alterations in the mucus layer [56]. In contrast to the small intestine, colonized by facultative anaerobic bacteria, the human colon is home to obligate anaerobic bacteria with high fermentative activity and proficiency to degrade mucins, such as *Akkermasia muciniphila* phylum Verrucomicrobia [57,58]. Moreover, the GM requires reduced pO_2_ (in the colon, less than 10 mmHg) and a hypoxic habitat for metabolism, hypoxia-inducible factor (HIF) transcription signaling, and proper autophagy of mucus and mitochondria [59]. For details on the mutual relationship between the intestinal barrier and microbiota, please refer to a recent review by Gierynska et al. [60].

## 3. Human Intestinal Microbiota in Health and Cardiovascular Diseases

### 3.1. Homeostatic Functions of Human Intestinal Microbiota

The human GM comprises over 100 trillion microbial cells, such as bacteria, fungi, viruses, and parasites. These microorganisms are dependent on the human gut and help the host to complete multiple physiological and biochemical functions, accompanied by their metabolites [61,62]. In a healthy bacterial community, the phyla Firmicutes and Bacteroidetes are the dominant flora, accounting for more than 90% of the population [63].

The host-specific composition is relatively stable over time [64,65,66]; however, the balance of bacterial species that can be beneficial and harmful to the host [67,68] can change throughout an individual’s lifetime in response to endogenous and exogenous factors. The relative abundance of bacterial species varies among individuals due to various genetic and environmental factors, including diet and antibiotic use [64,69]. The gut microbiota is necessary for the maintenance of host homeostatic functions through its involvement in fundamental processes, including the prevention of colonization by pathogens [70], detoxification of BAs [71], metabolism of non-digestible carbohydrates [72,73], and generation of crucial metabolites important for human health [74]. Due to their primary roles, we focus on these last two processes in more detail.

#### 3.1.1. Metabolism of Non-Digestible Carbohydrates

The gut microbiota have coevolved with us to serve a symbiotic role in extracting calories from otherwise indigestible macromolecules [68,75]. Colonic bacteria ferment indigestible carbohydrates and proteins to form short-chain fatty acids (SCFAs), volatile fatty acids containing fewer than six carbons, quickly and efficiently absorbed in the distal gut [76]. Butyrate, acetate, and propionate are the three most abundant SCFAs in the human colon (molar ratio of 60:20:20, respectively [77]). Acetate functions in fat regulation and storage, and most of it is oxidized by muscle or used by adipocytes for lipogenesis. At the same time, the remaining acetate is converted into butyrate by luminal bacteria [78,79,80]. Butyrate regulates gene expression in colonocytes and is essential in energy and glucose homeostasis [68,81]. Patients with inflammatory diseases show butyrate levels significantly lower than healthy controls [81,82]. On the contrary, propionate is taken up by the liver and is used as a substrate for gluconeogenesis [68,83]. Moreover, propionate has also been shown to stimulate the intestinal release of the satiety hormone peptide YY (PYY) and glucagon-like peptide 1 (GLP-1), leading to reduced energy intake in humans.

#### 3.1.2. Microbiota and the Regulation of Immune Responses

The role of the GM in maintaining host homeostasis also occurs through the constitution of the epithelial barrier and the regulation of the immune system.

GM products play a vital role in modulating immune responses, including those recognizing bacterial antigens and microbial metabolites. Intestinal epithelial cells detect bacteria and other microbes through TLRs and other pattern recognition receptors (PRR) expressed on immune cells, such as macrophages and DCs. These receptors recognize pathogen-associated molecular patterns (PAMPs) present on microbes to initiate an immune response [67,84].

Lipopolysaccharide (LPS) is localized in the outer membrane of Gram-negative bacteria, the most abundant bacteria in the gut microbiome [85]. The lipid A component of LPS represents the main PAMP that can interact with Toll-like receptor 4 (TLR4) [86,87,88], expressed on macrophages, endothelial cells (ECs), enterocytes, and DCs [88]. In response to LPS binding, TLR4 activates several signal transduction responses, resulting in the production of pro-inflammatory cytokines, such as TNF-α, interleukin-1 (IL-1), and interleukin-6 (IL-6) [87,89], as well as chemokines and cell adhesion molecules [88,90,91,92], which promote monocyte adhesion to the endothelial layer. For example, mice injected with LPS showed lower plasma HDL cholesterol and elevated plasma triglycerides [93]. This association was confirmed in humans.

A retrospective study conducted on 587 individuals from the Finnish Diabetic Neuropathy cohort revealed that patients with the highest serum LPS presented more elevated triglycerides in sera and higher blood pressure [94].

The GM is also necessary for dampening an immune response to non-pathogenic bacteria, thus protecting the host from the harm caused by sterile inflammation. Indeed, they contribute to developing the GALTs, in which adaptive immune cells undergo initial priming and differentiation [41,84,93,95]. The dynamic gut habitat needs heterogeneous, versatile, and convertible T cells, capable of inhibiting (Foxp3(+) T cells) or helping (T(FH) cells). It has been reported that the microbiota regulates the early-life B-cell repertoire generation in GALT and affects B-cell activation and differentiation to ultimately regulate B-cell function. Microbial antigens activate B cells directly via the BCR or TLRs. In contrast, microbial metabolites act directly on B cells to trigger their activation and differentiation into regulatory B cells, which produce regulatory cytokines or plasma cells that secrete anti-commensal antibodies; see the review in Yu et al. [96].

Other immune and nonimmune cells, such as DCs, serve as messengers for the GM activation of B cells indirectly through mechanisms, such as cytokine production or B-cell interactions.

Collectively, the microbiota plays an essential role in educating and shaping the host immune system, which, in turn, regulates GM diversity and function to maintain homeostasis. The homeostatic functions of the GM have close connections with each other. For example, besides being an energy source for both the host and microbiota, SCFAs are also signaling molecules that bind to G-protein-coupled receptors GPR41 and GPR43 [97], expressed in the adipose tissue, intestines, and immune cells [98]. GPR43 receptors are essential for neutrophil recruitment, and the interaction between SCFAs and GPR43 is crucial in regulating the inflammatory response [99].

### 3.2. Gut Dysbiosis and the Development of Cardiovascular Diseases

Gut dysbiosis is defined as an “imbalance of natural flora” in the GM composition [69]. When dietary habits, environmental factors, intestinal infection, or other factors lead to alterations in the species and quantity of intestinal microorganisms in the adult gut, gut dysbiosis occurs, causing inflammation and metabolic disorders [100]. The ratio of Firmicutes (F) and Bacteroidetes (B) (F/B) is considered a biomarker for gut dysbiosis [101].

Non-communicable diseases, such as diabetes [102,103], obesity [104,105], allergic asthma [106,107], and cancer [108], are increasingly associated with dysbiosis or changes in microbial composition. Furthermore, the central role of dysbiosis in the progression of atherosclerosis and hypertension, two major risk factors for CVDs, has been assessed. The recently discovered contribution of GM-derived molecules in the development of heart disease and its risk factors has significantly increased attention towards the close connection between the gut and heart [109,110].

In a study of patients with the highest and lowest lifetime burdens of CVD risk factors, respectively, a change in microbiota profile was found to be significantly associated with overtly increased risk [111]. Many microorganisms, such as *Chlamydophila pneumoniae*, *Porphyromonas gingivalis*, Influenza A virus, *Helicobacter pylori*, Cytomegalovirus, Hepatitis C virus, and human immunodeficiency virus, have been associated with an increased risk for CVDs [112].

The GM and its metabolites, such as SCFA, LPS, BAs, and trimethylamine-N-oxide (TMAO) impact cardiovascular health, thus being implicated in the onset or progression of hypertension and vascular damage [113]. The role of these GM products in promoting CV damage is described in the following paragraphs.

### 3.3. Atherosclerosis and Gut Microbiota

Several studies indicate that gut dysbiosis can contribute to the development and progression of atherosclerosis. Over 50 species of bacterial DNA have been observed in atherosclerotic plaques [114,115]. The presence of DNA from various species of bacteria in the atherosclerotic lesions and guts of the same individuals suggested that GM is a potential source of atherosclerotic plaque-resident bacteria and participates in the pathogenesis of coronary artery diseases [114,115]. It was identified that members of *Enterobacteriaceae* and *Streptococcus* spp. were more prevalent in atherosclerotic patients than in healthy controls [116,117]. Shotgun sequencing of the gut metagenome revealed that intestinal microbial communities in patients with symptomatic atherosclerosis, defined as carotid stenosis leading to cerebrovascular events, differed from those in healthy controls. Indeed, patients had an increased level of the genus *Collinsella*, while the gender- and age-matched controls had increased levels of *Eubacterium* and *Roseburia* [118]. A metagenome-wide association study on stools from 218 individuals with atherosclerosis and 187 healthy controls revealed an increased abundance of *Enterobacteriaceae* and *Streptococcus* spp. The GM collectively is less fermentative and more inflammatory in patients with coronary microvascular dysfunction [119].

Besides human patients, similar evidence in obese and hypercholesterolemic murine animal models has highlighted the role of the GM as a risk factor in the development of atherosclerosis [119,120]. Chan et al. analyzed atherosclerotic apolipoprotein E knockout (ApoE^−/−^) mice fed a high-fat diet for 12 weeks and supplemented with *Lactobacillus rhamnosus* GG or telmisartan [121]. Both supplements changed the GM composition and reduced the atherosclerotic plaque size. Similarly, another five bacterial species (*Eubacteria*, *Anaeroplasma*, *Roseburia*, *Oscillospira*, and *Dehalobacteria*) prevented atherosclerosis. In contrast, *Porphyromonas gingivalis* and *Aggregatibacter actinomycetem comitans* were associated with the acceleration of atherosclerosis in animals after dietary intervention or intravenous infusion [122]. Finally, the absence of microbiota could cause an increase in atherosclerotic lesions compared with conventionally raised controls [123].

#### Role of Trimethylamine-N-Oxide, LPS, and Bile Acids in Atherosclerosis

Two major pathways have been highlighted to provide a mechanistic description of the role of dysbiosis in atherosclerosis.

The first one is related to the increased gut permeability induced by dysbiosis. The LPS/TLR4-mediated production of pro-inflammatory cytokines promotes monocyte adhesion to the endothelial layer. These are called foam cells, a major component of atherosclerotic plaques. Foam cells are macrophages, phagocytic immune cells that engulf excessive amounts of modified low-density lipoprotein (LDL) cholesterol to remove it from the bloodstream [124,125]. Reverse cholesterol transport (RCT) is a homeostatic mechanism by which cholesterol in excess is converted in the liver into BAs [126,127,128]. Gut dysbiosis can overwhelm RCT functions and promote the formation of foam cells [129,130,131,132].

The second mechanism is the metabolism-dependent pathway, whereby dysbiosis exerts pro-atherosclerotic effects by altering the generation of various metabolites [133]. BAs are synthesized from cholesterol; their synthetic pathway is a major route for cholesterol elimination. The GM can catalyze the deconjugation of primary BAs within the intestinal lumen to form secondary ones through bacterial bile salt hydrolase (BSH) [50]. In dysbiosis, there is decreased BSH activity, leading to the accumulation of cholesterol, the formation of foam cells, and, ultimately, to the atherosclerotic plaque [131]. Notably, butyrate, one of the primary SCFAs produced by the bacterial fermentation of non-digestible carbohydrates, has athero-protective and anti-inflammatory effects, reducing monocyte adhesion to the endothelium [103] and the expression of vascular cell adhesion protein 1 (VCAM-1), hindering foam cell formation [134,135].

Besides changes in BA metabolism, GM alteration contributes to the production of atherosclerotic TMAO in the gut.

TMAO is a metabolite derived primarily from dietary phosphatidylcholine and L-carnitine. First, phosphatidylcholine and other TMA-containing compounds, such as L-carnitine, are metabolized by bacterial enzymes TMA lyases to produce the gas trimethylamine [109]. Then, TMA enters the liver through the portal circulation and is oxidized into TMAO by hepatic flavin monooxygenases [110,136,137].

Metabolomics first identified TMAO and choline as small-molecule metabolites associated with CVD risk in human plasma. Furthermore, the increased occurrence of heart failure has been directly linked to higher baseline levels of TMAO, demonstrating the significance of this marker as a predictor of cardiovascular risk [138].

Li et al. revealed that the TMAO level in acute coronary syndromes was an independent predictor of short-term (30-day and 6-month) and long-term (7-year) adverse cardiac events [139]. Other studies highlighted the participation of TMAO in the development of CVDs in a variety of patient cohorts [140,141,142]. Conversely, only one study reported that TMAO could reduce cholesterol reabsorption and was beneficial against atherosclerosis [143].

TMAO promotes the development of atherosclerosis by inhibiting RCT and cholesterol catabolism and, consequently, increases foam cell formation, thus accelerating atherosclerosis [109,110,144]. Additionally, TMAO can reduce cholesterol clearance from the body by decreasing the expression of the hepatic BA synthetic enzymes and inhibiting BA synthesis [145,146].

### 3.4. Metabolic Syndrome and Gut Microbiota

Insulin resistance and inflammation are the underlying causes of metabolic syndrome (MetS). High blood triglycerides, altered cholesterol levels, glucose intolerance, and hypertension greatly increase the risk of type 2 diabetes and CVDs [147]. While the GM is responsive to large caloric intake fluctuations, multiple studies show that it is particularly sensitive to diet composition [148,149]. The first human trial involving oral *Akkermansia muciniphila* supplementation in overweight/obese insulin-resistant individuals significantly ameliorated insulin sensitivity and reduced insulinemia and total plasmatic cholesterol [150]. Dyslipidemia represents an abnormal amount of lipids in the blood. Dyslipidemia and the resulting atherosclerotic plaques are major CVD risk factors, often intricately linked with impaired glucose metabolism and obesity [151].

Profiling of the GM of 531 well-phenotyped Finnish men from the Metabolic Syndrome in Men study revealed several associations between the GM and MetS. SCFAs produced by the GM affected insulin sensitivity and suppressed insulin-mediated fat accumulation [98]. SCFAs also regulate energy intake by stimulating the secretion of satiety hormones GLP1 and PYY [152,153]. Intriguingly, fecal microbiota transplants from lean donors to insulin-resistant MetS individuals increased insulin sensitivity and the number of microbiota-producing butyrate, an SCFA known to affect satiety hormones [154].

### 3.5. Hypertension and Gut Microbiota

Hypertension is the most important modifiable risk factor for CVDs [155]. Indeed, less than 5% of the incidence of hypertension can be explained by genetics [156], whereas non-genetic factors, such as body mass index (BMI) and salt intake, tend to have a prominent role [157]. In addition, several dietary interventions have illustrated that a higher intake of vegetables, fruits, and fiber is associated with a reduction in blood pressure [158,159].

The GM could affect hypertension through inflammatory factors influenced by SCFAs and LPS. Several studies have reported compositional differences in the GM in animal models for hypertension compared to wild-type animals [160,161,162,163]—for example, a lower abundance of SCFA-producing bacteria, a higher abundance of lactate-producing bacteria, a lower abundance of *Bacteroidetes*, and a higher abundance of *Proteobacteria* and *Cyanobacteria* [163]. Blood pressure levels in hypertensive animal models could be modified by fecal microbiota transplants and antibiotic treatment [162]. Moreover, fecal SCFA concentrations in humans have been associated with higher blood pressure [164], while SCFA-producing microbiota have been associated with lower blood pressure [165]. Indeed, increased SCFA availability in the intestines upregulates absorption mechanisms, leading to relatively lower fecal concentrations and higher plasma availability [162]. It is known that the Mediterranean diet, which induces a rise in SCFA levels, has a blood pressure-lowering effect [166].

Several cross-sectional studies on the associations between GM composition and blood pressure or hypertension indicated that microbial abundance, diversity, and evenness decreased in spontaneously hypertensive rats but also in human patients due to high blood pressure [165,167]. In addition, a higher concentration of Gram-negative bacteria, including *Klebsiella*, *Parabacteroides*, *Desulfovibrio*, and *Prevotella*, was associated with higher blood pressure. Gram-negative bacteria are a source of LPS. In contrast, SCFA-producing bacteria, including *Ruminococcaceae*, *Roseburial*, and *Faecalibacterium* spp., were higher in normotensive individuals than in hypertensive ones [139,165,167]. Dietary salt intake also affects the GM composition, and higher salt intake induced an increase in *Lachnospiraceae*, *Ruminococcus*, and *Parasutterella* spp. and a decrease in *Lactobacillus* and *Oscillibacter* in animal models [167,168,169,170].

The effects of SCFAs on blood pressure are different depending on the receptors involved. SCFAs, including butyrate, have anti-inflammatory effects, probably mediated by the inhibition of histone deacetylase (HDAC) [171,172]. Butyrate added to in vitro monocytes suppresses the production of pro-inflammatory cytokines, such as tumor necrosis factor-α (TNF-α), interleukin-12 (IL-12), and interferon-γ (IF-γ), and upregulates the production of anti-inflammatory interleukin-10 (IL-10) [173]. In addition, SCFAs have anti-inflammatory effects on epithelial cells that are partly mediated through HDAC [174].

Gut dysbiosis contributes to hypertension through oxidated LDL (oxLDL)-induced vasoconstriction and promotes pro-inflammatory cytokine expression and foam cell formation [175]. Inflammation induces oxidative stress, and vice versa, sustaining a positive feedback loop that promotes an increasingly oxidative environment [176]. Elevated oxidative stress can stimulate oxLDL, which causes the underproduction of vasodilators and the overproduction of vasoconstrictors, leading to hypertension [175,177,178].

Moreover, LPS-related gut permeability has been associated with a hypertensive state. Animal studies suggest that systemic LPS could have pro-inflammatory, sympathetic activating, and neuroinflammatory effects relevant to hypertension pathogenesis. Indeed, LPS administration to rats enhanced the heart rate, norepinephrine levels, and neuroinflammation, indicated by sustained TLR and TNF-alfa expression in the paraventricular nucleus, the regulator of blood pressure [179]. Hypertensive rats had lower mRNA levels of gap junction proteins, indicating higher gut permeability, restored after fecal microbiota transplantation from healthy controls. Similarly, in spontaneously hypertensive rats, increased blood pressure was associated with more permeability and lower levels of tight junction proteins [180].

**Figure 2:**
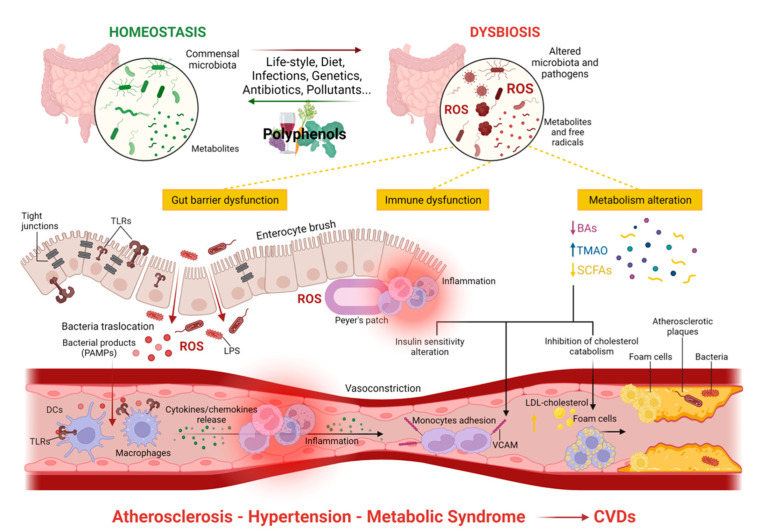
The role of gut microbiota alterations in the main mechanisms associated with risk factors for CVDs.

Overall, it has emerged that multiple adverse cardiovascular events—mainly atherosclerosis, metabolic syndrome, and hypertension—can be linked to GM dysbiosis. The association between CVD risk factors (atherosclerotic plaque instability), as well as gut barrier and immune dysfunctions, and metabolism alterations due to GM dysbiosis are represented in Figure 2.

## 4. Polyphenols in the Diet: Classification and Bioavailability

Polyphenols represent a large class of bioactive compounds whose chemical structure is characterized by a polyphenol skeleton in which one or more aromatic rings are present and linked to one or more hydroxyl (-OH) groups.

This class of plant-derived compounds is one of the most numerous and widely distributed groups of phytochemicals: the main sources include vegetables, whole grains, fruits, and several beverages, such as chocolate, tea, beer, and wine. More than 8000 polyphenols have been identified and may vary significantly in terms of chemical structure, bioavailability, stability, and physiological function related to human health [181]. Indeed, polyphenolic compounds can vary from simple to highly polymerized molecules, and, based on the number of phenol rings, their arrangements, and the structural components connecting such rings, they are classified as flavonoids and non-flavonoids, with the latter referring specifically to phenolic acids, stilbenes, coumarins, and lignans [182,183,184].

### 4.1. Flavonoids

**Figure 3:**
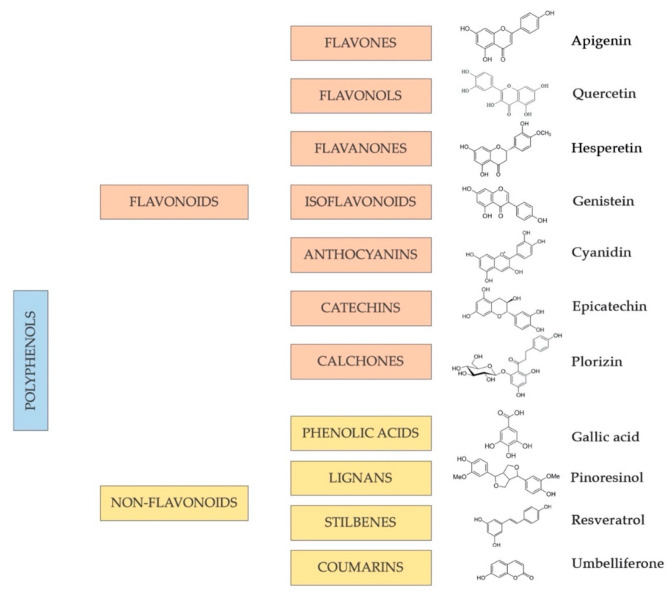
Phenolic compound scheme: classes, sub-classes, and molecular structure of a typical compound of each sub-class.

Polyphenols classified as **flavonoids** include several natural, low-molecular-weight phenolic compounds widely distributed in the plant kingdom. In particular, flavonoids can be described as plant secondary metabolites, also abundantly found in beverages and foods of plant origin, such as vegetables, fruits, grains, stems, roots, tea, wine, and cocoa; for this reason, they are termed dietary flavonoids [185]. From this perspective, flavonoids are considered the most abundant dietary polyphenols, representing approximately two-thirds of all phenolic compounds ingested in the human diet [186,187]. The sub-class of flavonoids groups together several compounds that have a common basic chemical structure/skeleton: two benzene rings are connected by a three-carbon bridge. The benzene rings are termed ring A and B, while the three-carbon chain that links ring A with ring B is named ring C, a heterocyclic and oxygen-containing ring (Figure 3). Depending on the degree of oxidation and saturation of ring C, and to which carbon the ring B is attached, they can be subdivided into diverse subclasses: flavones, flavanols, flavanones, flavanonols, isoflavones, catechins, anthocyanidins, and chalcones. Each of them has its major sources. Most flavonoids are naturally glycosides rather than aglycones [188].

### 4.2. Non-Flavonoids

With some exceptions [187], the phenols categorized as **non-flavonoids** (phenolic acids, stilbenes, coumarins, and lignans) are compounds with a simpler and smaller chemical structure compared with that of flavonoids [189]. This sub-class of compounds is characterized by a greatly heterogeneous chemical structure that is associated with their definition (Figure 3).

(i) **Phenolic acids** usually include phenolic compounds having one carboxylic acid group [181]. Depending on their origin—whether derivatives of benzoic acid or cinnamic acids—phenolic acids can be differentiated into two classes: hydroxybenzoic acids and hydroxycinnamic acids. Hydroxybenzoic acids are the simplest phenolic acids, although they are less common: they are usually found in soluble form (glycosylated) [190] and in low concentrations in vegetables and fruits, such as onions and red fruits [184]. Differently, hydroxycinnamic acids, which are more common in plants than benzoic acid derivatives [191], include coumaric acid, ferulic acid, caffeic acid, sinapic acid, and rosmarinic acid, frequently found in several foods, including coffee.

(ii) **Coumarins,** a sub-group of phenolic compounds derived from *o*-cumaric acid [192,193], can be found in free or glycosylated form. Coumarin, umbelliferon, esculetin, and scopoletin are coumarin derivatives found in olive oil, aromatic herbs, and spices [192].

(iii) **Lignans** are low-molecular-weight phytoestrogens found, in low concentrations, in plants, particularly whole grains, nuts and seeds, soybeans, and cruciferous vegetables [194]. Among lignans, secoisolariciresinol diglycosidic is an essential dietary example that, when consumed, is converted by intestinal bacteria into enterodiol and enterolactone with weak estrogenic activity [195].

(iv) **Stilbenes** are natural defense phenolic compounds abundant in many plant species, including peanuts, berries, grapes, and *pinus* species, present in the human diet [196]. Resveratrol is the most important and studied stilbene [197] due to its well-known anti-aging and antioxidant activity, found in peanuts, cocoa, and particularly in grapes and wines. Resveratrol was also demonstrated to be effective in the treatment of tumors, such as glioma [198]. However, other stilbenes have attracted greater attention due to their health-beneficial properties, such as pinosylvin and pterostilbene [199,200].

The most representative polyphenols and their main dietary sources are presented in Table 1 and Table 2.

### 4.3. Low Bioavailability/High Bioactivity Paradox of Polyphenols

The existence of a wide and heterogenic range of phenolic compounds (Figure 3) may obviously influence their course and properties in humans once ingested with foods and beverages. Their metabolism course and deriving metabolites, biological activities and health-beneficial properties, target tissues, and bioavailability are still controversial, despite the fact that this class of compounds has received widespread interest in the last several decades.

Overall, polyphenols provide health benefits for humans thanks to their major antioxidant action in target tissues, but the first condition to accomplish such actions is their bioavailability [225,226].

Bioavailability refers to the rate and extent to which a drug or substance becomes available to its biological destination, i.e., the systemic circulation or tissue/organ targets [227,228]. It can be widely influenced by a series of variables: metabolic steps, specificity of target receptors, administration and absorption, and possible interaction with other substances or transformation by intestinal microflora. Noticeably, the compounds’ molecular diversity has a major influence on their bioavailability.

Bioavailability comprises the absorption phase, which is one of the main limiting factors, followed by the compound’s liberation from the food matrix, distribution, metabolism, and elimination [229]. Hence, its metabolism is crucial to determine which polyphenol is better absorbed and produces bioactive metabolites.

After ingestion, the absorption of dietary polyphenols and monomeric and dimeric structures (such as O-glycosides) occurs in the small intestine (duodenum and jejunum), where they arrive intact (5–10% of total intake)—with the exception of anthocyanidins, usually degraded from glycosidase enzymes by the oral microflora [230]. Upon arrival in the small intestine, the intact glycoside form is converted through a hydroxylation reaction into the aglycone form by the enzymes expressed in the GM (β-glucosidase CBG and lactase-phlorizin hydrolase LPH enzymes) [231,232]; it is then absorbed by enterocytes and moved to the liver through the portal vein [233]. The intestinal absorption for compounds with low molecular weight (such as isoflavones and gallic acid) and high lipophilicity (as aglycones) may occur by passive diffusion or via transporters [189]. In other cases, such as for quercetin glycosides, to allow their entry within enterocytes, cotransporters may be required [234]. Differences in absorption are due to their chemical characteristics [235,236,237]. Interestingly, the remaining large number of polyphenols (90–95% of the total), namely high-molecular-weight oligomeric and polymeric polyphenols, pass unchanged to the small intestine and reach the colon, where they will be absorbed after hydroxylation by GM-secreted enzymes (such as the α-rhamnosidases) [238]. Indeed, the GM shapes the original structures of complex polyphenols into low-molecular-weight metabolites that will then become absorbable and bioactive metabolites [239].

Prior to entry into the bloodstream, polyphenols undergo other structural modifications, mainly in the liver. Depending on the chemical structure of each absorbed phenolic compound, hydroxylation, thiolation, carboxylation, glucuronidation, methylation, and sulfation, or a combination of them, are all examples of the multiple reactions possibly occurring in the liver [240,241].

Polyphenol metabolites’ destinations may be diverse and include the brain, pancreas, lungs, spleen, and heart. Polyphenols are considerably modified [242], and these modifications generate several metabolites, from two or three for most of the compounds to as many as 20 in the case of quercetin glycosides [243]. Consequently, polyphenolic compounds in the bloodstream are chemically different from the original dietary form.

Isoflavones and phenolic acids, such as caffeic acid and gallic acid are the most well-absorbed polyphenols, followed by catechins, flavanones, and quercetin glucosides [186], while polyphenols with a large molecular weight (such as proanthocyanins, galloylated tea catechins, and anthocyanins) are considered the least absorbed polyphenols [240]. Recent studies in the food industry on the delivery of green tea catechins (mainly epigallocatechin gallate, EGCG), through liposomal encapsulation or nanoencapsulation in functional foods, indicate new avenues to improve their poor stability and absorption in the upper intestine [244].

However, despite the increased amount of data available, definitive conclusions on the bioavailability and bioactivity of a single phenolic compound are still difficult to obtain. The variability in dietary habits and the GM among individuals may produce great differences in polyphenols’ bioavailability. For example, the production of active metabolites from isoflavones after soybean consumption has been estimated at 30% in the urine in an occidental population [245], while it was doubled (60%) in a Japanese population [246].

## 5. Antioxidant and Anti-Inflammatory Effects of Polyphenols on Cardiovascular System

Regardless of the bioavailability and low absorption of dietary polyphenols, these compounds possess a wide range of beneficial biological activities, mainly antioxidant and anti-inflammatory properties [247,248,249]. Free radical scavenger activity, mitochondrial protection, transcription factor regulation, membrane receptor modulation, ROS inhibition, and anti-proliferation are all mechanisms exerted by polyphenols and are greatly described in the literature [34,250]. These beneficial effects can be exploited in preventing and treating acute and chronic diseases, such as metabolic disorders, cancer, inflammation, neurodegeneration, and CVDs [251,252].

Dietary regimens based on polyphenol-rich foods greatly correlate with reduced morbidity and a milder course of CVDs [253]. A recent study by the Optimal Nutraceutical Supplementation in Heart Failure (ONUS-HF) group confirmed the potential of the combination of natural products, such as apple-derived phenolic-glucoside phlorizin, *Vitis vinifera* extracts, bergamot polyphenolic fraction, and *Olea Europea* L-derivatives in patients at an early phase of myocardium failure [254]. Cardiovascular-protective mechanisms induced by polyphenols firstly rely on their potent antioxidant properties, which may explain their beneficial effects on a wide range of related comorbidities. Their role as inhibitors of oxidative stress is ascribed to the presence of hydroxyl groups in their chemical structure, which are promptly oxidated: ergo, an electron or H atom donated from the aromatic hydroxyl group neutralizes a free radical [251]. This conversion generates stabilized chemical structures that entrap free radicals, producing effective scavenger activity, thus preventing further reactions [252]. This direct antioxidant property leads to the scavenging of free radicals, such as ROS, reactive nitrogen species, hypochlorous acid, and NO, and the products of the peroxidation of lipids, proteins, and DNA [181,251].

However, although the inhibition of oxidative stress by polyphenols has been proven in several in vitro experiments [252], consistent data obtained by in vivo experiments are scarce; thus, evidence supporting their direct antioxidant activity in vivo is still weak [255]. There are doubts regarding the pro-oxidative activity of phenolic compounds in vivo due to their low plasma bioavailable concentration once ingested, low bioavailability, poor absorption, rapid metabolism, and poor stability. Moreover, the antioxidant capacity of these compounds decreases over their ‘journey’ through the human gastrointestinal tract: their metabolism produces modifications of the original chemical structure and the resulting products sometimes have their -OH groups blocked by several transformative processes, compromising or reducing their potential antioxidant capacity [251,256].

Another beneficial mechanism hypothesized for polyphenols concerns their property in influencing inflammation and, consequently, as described above, in the process and progression of atherosclerosis [257,258]. For instance, findings of some in vitro and animal studies revealed that quercetin and resveratrol play a determinant role in influencing inflammation [259]: their consumption was proven to reduce inflammation, with a consequential attenuation of lipid peroxidation, cholesterol regulation, and platelet aggregation and a reduction in atherosclerotic plaque progression. In other words, one potential mechanism implemented by phenolic compounds is likely to modulate the transcriptional network and signaling cascade to reduce pro-inflammatory mediators and VCAM-1 in the endothelium, with the final consequence of suppressing the migration of monocytes into the subendothelial space [259,260].

The pleiotropic properties displayed by polyphenols in the cardiovascular system are various, including vasodilator, antiatherogenic, antithrombotic, antiapoptotic, hypolipemic, and anti-inflammatory effects, all associated with a reduction in cardiovascular risk. These properties reflect the ability of phenolic compounds to participate in different metabolic cellular oxidative reduction reactions, and in the modulation of enzyme actions and signaling mechanisms [30,261].

However, although a broad consensus exists on the beneficial effects exerted by polyphenols on the cardiovascular system, no singular mechanism of a specific polyphenol compound has been directly correlated in vivo to the improvement of endothelial health, and the prevention of hypertension and cardiovascular diseases. On the contrary, the cardiovascular benefits of plant-based rich polyphenols may likely depend both on the quantity and reciprocal interactions of polyphenolic compounds, acting through several pathways, leading to a healthy synergistic action [262].

## 6. Polyphenol–Gut Microbiota Interaction in Cardiovascular Diseases

Bioactive metabolites derived from a polyphenol-rich diet are strictly correlated to the individual’s GM capabilities and genetic profile [263]. Although the true extent is still undetermined, the cardiovascular protection activity affected by circulating bioactive phenolic metabolites is undoubted; Villa-Rodriguez et al. [264] suggest that the gastrointestinal tract might represent a prime site for cardioprotection by polyphenols. Importantly, in addition to the numerous direct health-beneficial effects ascribed to dietary polyphenols, these compounds may influence/modulate the activity and composition of the GM [261]. The GM is believed to have a reciprocal interaction with polyphenols: once the phenolic compounds arrive in the gut, mainly in the colon, they regulate the local redox state, increasing the production of bioactive metabolites and favoring the growth of beneficial bacteria, carrying out their so-called ‘prebiotic effect’ [265]. In these terms, the microbiota represents a key link between the health-beneficial effects produced by polyphenols and metabolic and chronic diseases [250]. Many bacterial species are involved in the metabolism of polyphenols: *Flavonifractor plautii*, *Slackia equolifaciens*, *Slackia isoflavoniconvertens*, *Adlercreutzia equolifaciens*, *Eubacterium ramulus*, *Eggerthella lenta*, and *Bifidobacterium* spp. These are all examples of microflora contributing to the generation of circulating bioactive metabolites with positive effects on health.

The mechanisms of action through which polyphenols modulate the GM composition are still unclear, but the regulation of bacterial multiplication is likely one of the examples: the GM’s growth can be both inhibited and activated by phenolic compounds [266]. The two effects can be directly induced, or one may be the consequence of the other: there can be direct inhibition of the growth of one specific bacterium, a direct increase in a specific bacterial population, or an indirect reduction in the growth of one bacterial species due to a direct increase in the development of another bacterial population [266]. However, regardless of the type of action that each phenolic compound can exert in the microbiota composition, polyphenols can enhance the presence of beneficial bacteria and reduce the growth of pathogenic species. They can, for instance, augment the genera correlated with anti-inflammatory (*Faecalibacterium*) and gut barrier protection (*Lactobacillus and Bifidobacterium*) effects, as well as other health properties. Several studies investigated the correlation between GM changes and the administration of a diet based on plant-derived polyphenol-rich foods: an increase in the genera of *Bifidobacterium* in the GM was proven after the general consumption of fruits and vegetables [267], *Schisandra chinensis* fruit [268], red wine [269], and cocoa drinks [270]. An analogous augmentation of the other favorable microbiota genera, namely *Lactobacillus*, was found too [266]. In addition, a recent study reported changes in the bacterial populations of the gut caused by several bioactive phenolic metabolites, which increased *Enterococcus*, *Bacteroides*, and *Prevotella* spp. [253]. However, depending on the type of polyphenol, some bioactive phenolic compounds, such as those contained in tea, can also have antimicrobial implications, leading to the reduction or suppression of several pathogenetic genera, including *Clostridium* [271], *Helicobacter pylori* [272], *Staphylococcus aureus* and *Escherichia coli*, *Salmonella typhimurium*, and *Listeria monocytogenes* [39]. Patients with chronic heart failure present gut alterations, which contribute to a vicious cycle based on decreased absorption and enhanced inflammation, due to hypoxia in the intestine [273]. ‘Leaky gut’ due to abnormal microcirculation is a hallmark of a disrupted intestinal barrier, with increased permeability to GM toxins associated with metabolic damage and inflammation [274,275].

The heart–gut axis might be a novel target for prognosis and treatment in CVDs [276,277]. However, whether the *gut hypothesis* of heart failure is the cause or the consequence of cardiac damage is unclear [278]. In this scenario, dietary polyphenols, by modulating the GM and GM metabolites, might positively influence human health [279]. Consumption of dietary polyphenols for 8 weeks in geriatric subjects in a nursing home alleviated the altered intestinal permeability and bacterial products in the circulation [280]. The same authors reported that bacterial DNA, mainly Proteobacteria and *Pseudomonas* genera, tested in the blood in older voluntary subjects, decreased after consumption of a polyphenol-rich diet. Consequently, systemic inflammation and intestinal barrier composition were ameliorated, suggesting that DNAemia may be a relevant marker for selecting more vulnerable populations with a higher cardiovascular risk [281]. Kiwi fruit polyphenolic extract reduced colon permeability, increased the number of *Bacteroidetes*, *Lactobacillus*, and *Bifidobacterium*, and inhibited TLR inflammation in high-fat-diet-fed rats [282]. Whether the GM may be a new druggable target to predict and treat CVDs is a fascinating research opportunity that requires further study.

The multitude of *in vitro*, *in vivo*, and animal studies whose findings have been only briefly mentioned here makes it difficult to obtain a clear conclusion on the direct benefits that dietary polyphenols might provide in heart diseases. Hence, as the literature on this topic has exponentially increased over the past 10 years, and clinical data in patients sometimes are contradictory and incongruent, we are still far from verifying these results with unequivocal conclusions without any speculations. However, it seems clear that, besides *Bifidobacterium* and *Lactobacillus*, bioactive phenolic compounds exert positive modulatory effects on other gut microbes, which in turn are proven to affect markers mainly associated with CVDs. This is supported also by two recent studies that indicated the existence of a gut–heart axis able to influence cardiovascular adverse events and their clinical biomarkers [283,284,285]. In particular, it has been reported that intestinal cells where the GM is affected produce low amounts of proprotein convertase subtilisin/kexin type 9 (PCSK9), a crucial enzyme involved in cholesterol dismantling via LDL. Indeed, low PCSK9 is a marker of atherosclerosis, and the restoration of proper bacteria colonization greatly impacts cardiovascular health.

## 7. Conclusions: Is There a Gut–Heart Axis Modulated by Polyphenols?

An abnormal GM is common in aged patients hospitalized for heart failure, often receiving more than 10 different medications per day. For this type of patient, an attempt to limit polypharmacy and substitute or integrate drugs with natural plant-derived foods may be relevant to mitigate adverse drug side effects [286]. Moreover, modern lifestyle habits, with a diet rich in fat and low in fiber, are associated with an increase in the incidence of diseases, including CVDs, related to the dysregulation of the intestinal bacterial flora. For these reasons, understanding the mechanisms by which polyphenols can improve cardiovascular function could be crucial for the treatment and prevention of CVDs. A recent animal study in hypercholesterolemic ApoE^−/−^ mice treated with antibiotics affecting the GM demonstrated that the common statin therapy could become ineffective [287]. Whether dietary polyphenols, in single or combined formulations, could treat cardiovascular failure directly and indirectly by modulating the GM is still an open question. The beneficial role of polyphenols towards gut symbiosis and prebiotic effects, as well as antimicrobial activity against pathogenic microflora (both factors exacerbating healthy impacts on the cardiovascular system), needs more experimental and consistent evidence supported by clinical research. The recent biotechnological production of oral formulations of polyphenols in nanocapsules added to foods and beverages, strengthens their delivery in order to treat cardiovascular damage [288]. However, more in-depth mechanistic knowledge is required for a better understanding of the molecular basis behind polyphenol efficacy in the gut–heart axis, their proper dosage, the absence of side effects, and the necessity of safe formulations that are globally accepted, in addition to the need to obtain unambiguous outcomes from rigorous clinical trials in hospitalized patients. The pathway to the clinical application of dietary polyphenols to promote cardiovascular health through the GM is still at its beginning.

## Figures and Tables

**Table 1:** Sub-classes of flavonoid phenolic compounds, examples of compounds for each different sub-class, and their main dietary sources.

Sub-Class	Compounds	Major Sources
**Flavonols**	Quercetin Myricetin Rutin Morin Kaempferol	Onions; broccoli; tea; red wine; grapes; berries; lettuce; tomatoes; apples [201,202,203]
**Flavanones**	Hesperetin Eridicytol Naringenin	Citrus fruits; grapes [204,205,206]
**Flavones**	Sinsensetin Diosmetin Tangeretin Apigenin Luteolin	Citrus fruits; mint; olive oil; honey; vegetables of sunflower family; cereals and legumes; dry herbs; tea [207,208]
**Isoflavonoids**	Daidzein Genistein Glycitein	Soy and derivates; legumes [209]
**Anthocyanidins**	Cyanidin Delphinidin Peonidin Malvidin	Berries; grapes; cereals; beans; cabbages; onions; aubergines [210,211,212]
**Catechins**	Epicatechin Epigallocatechin	Tea; chocolate; red wine; apples; peaches; apricots; cherries; berries; beans [213,214,215]
**Chalcones**	Phlorizin Arbutin Chalconaringenin Phloretin	Tomatoes; berries; cereals (wheat products); pears; apples; tea [216,217]

**Table 2:** Sub-classes of non-flavonoid phenolic compounds, examples of compounds for each sub-class, and their main dietary sources.

Sub-Class	Compounds	Major Sources
**Stilbenes**	Resveratrol Phytoalexins Piceatannol	Grapes; berries; red wines; peanuts; cocoa [218,219]
**Phenolic Acids**	Gallic Acid Benzoic Acid Cinnamic Acid Caffeic Acid Ferulic Acid	Berries; red fruits; onions; black radish; whole grains and wheat; leafy and stem vegetables; barley; coffee; red wine; beer [220,221,222]
**Lignans**	Pinoresinol Lignin Silymarin Magnolol	Sesame; flax seeds; legumes; whole grain cereals; Brassica family vegetables [223]
**Coumarins**	Scopoletin Aesculetin Fraxin Umbelliferone	Grapes; olive oil; spices; aromatic plants [224]

## Data Availability

Not applicable.

## Abbreviations

ApoE^−/−^	Atherosclerotic apolipoprotein E knockout
B	Bacteroidetes
Bas	Bile acids
BMI	Body mass index
BCR	B-cell receptors
BSH	Bile salt hydroxylase
CRP	C-reactive protein
CVDs	Cardiovascular diseases
DCs	Dendritic cells
DNA	Desoxyribonucleic acid
ECs	Endothelial cells
EGCG	Epigallocatechin gallate
F	Firmicutes
FMO3	Flavin monooxygenase
FXR	Farnesoid X receptor
GALTs	Gut-associated lymphoid tissues
GLP-1	Glucagon-like peptide 1
GM	Gut microbiota
GPRs	G-protein-coupled receptors
HDAC	Histone deacetylase
HDL	High-density lipoprotein
HIF	Hypoxia-inducible factor
H&E	Hematoxylin and Eosin
IECs	Intestinal epithelial cells
IF-γ	Interferon-γ
IgA	Immunoglobulin A
IL-1,6,10,12	Interleukin 1,6,10,12
ISCs	Intestinal stem cells
LDL	Low-density lipoprotein
LPH	Lactase-phlorizin hydrolase
Lp	Lamina propria
LPS	Lipopolysaccharides
MetS	Metabolic syndrome
MM	Muscularis mucosae
NF-kB	Nuclear factor kappa-light-chain enhancer of activated B cells
NLRP3	Nucleotide-binding oligomerization domain leucine-rich repeat containing protein 3
NO	Nitric oxide
NOXs	NADPH oxidase
oxLDL	Oxidated LDL
ONUS-HF	Optimal Nutraceutical Supplementation in Heart Failure
PAMPs	Pathogen-associated molecular patterns
PCSK9	Proprotein convertase subtilisin/kexin type 9
PRR	Pattern recognition receptor
PPs	Peyer’s patches
PYY	Pancreatic peptide YY
PVN	Paraventricular nucleus
RCT	Reverse cholesterol transport
RNS	Reactive nitrogen species
ROS	Reactive oxygen species
SCFAs	Short-chain fatty acids
TGR5	Takeda G-protein-coupled receptor 5
TLRs	Toll-like receptors
TMA	Trimethylamine
TMAO	Trimethylamine-N-oxide
TNF-α	Tumor necrosis factor alpha
TJs Tight	junctions
VCAM-1	Vascular cell adhesion protein 1
WHO	World Health Organization
